# Characterizing heterogeneity in the response of synovial mesenchymal progenitor cells to synovial macrophages in normal individuals and patients with osteoarthritis

**DOI:** 10.1186/s12950-016-0120-9

**Published:** 2016-04-06

**Authors:** Akash Fichadiya, Karri L Bertram, Guomin Ren, Robin M Yates, Roman J Krawetz

**Affiliations:** McCaig Institute for Bone and Joint Health, University of Calgary, Cummings School of Medicine, Calgary, Canada; Department of Comparative Biology and Experimental Medicine, University of Calgary, Faculty of Veterinary Medicine, Calgary, Canada; Department of Surgery, University of Calgary, Cummings School of Medicine, Calgary, Canada

**Keywords:** Mesenchymal progenitor cell, Chondrogenesis, Macrophage, Synovium, Osteoarthritis

## Abstract

**Background:**

Resident macrophages in OA synovial tissue contribute to synovitis through pro-inflammatory mediators driving cartilage loss. What remains unknown is how these macrophages interact with synovial mesenchymal progenitor cells (sMPCs) in the joint. sMPCs have the potential to undergo chondrogenesis, but for yet unknown reasons, this ability is decreased in OA patients. In this study, we sought to identify if alteration of macrophage activity regulates the chondrogenic capacity of sMPCs.

**Methods:**

An explant model was developed using human synovium obtained from normal individuals and OA patients. These explants were subjected to macrophage depletion and/or cytokine stimulation in order to regulate/deplete the residing macrophage population. Supernatant was collected following a 12-day treatment phase and subjected to inflammatory secretome analysis. sMPCs from the explants were subsequently placed under 21-day chondrogenic differentiation and levels of type II collagen (Col2a), Aggrecan (Acan), and Sox9 gene expression was quantified.

**Results:**

Inflammatory secretome analysis from OA patients revealed the presence of pro-inflammatory analytes following pro- and anti-inflammatory cytokine stimulation and/or macrophage depletion. Additionally, chondrogenic differentiation of sMPCs was heterogeneously impacted across all OA patients following pro-/anti-inflammatory cytokine stimulation and/or macrophage depletion.

**Conclusion:**

Tissue resident synovial macrophages can regulate the chondrogenic differentiation of sMPCs after cytokine stimulation in a patient specific manner. The secretion profile of OA synovium was also responsive to cytokine stimulation and/or macrophage depletion as observed by the largely pro-inflammatory milieu upregulated following cytokine stimulation.

**Electronic supplementary material:**

The online version of this article (doi:10.1186/s12950-016-0120-9) contains supplementary material, which is available to authorized users.

## Background

The 2001 discovery of resident mesenchymal progenitor cells within the synovial membrane (sMPCs) of the joint presented an opportunity to understand the limited self-renewal and healing capacity of articular cartilage [1, 2]. Mesenchymal progenitors have the potential to differentiate into bone, fat, and importantly cartilage. However, in diseases such as osteoarthritis (OA), this chondrogenic phenotype appears to be modified for yet unknown reasons [3, 4]. This observation is of great interest given the potential cell-based therapies that can be derived using resident sMPCs. Interestingly, it has also been observed that inflammation of encompassing synovium (synovitis) and corresponding intimal hyperplasia, subintimal macrophage infiltration and increased vascularity are not observed without involvement of cartilage disturbance in OA – thereby implicating the synovium in disease pathogenesis [5–9]. This raises particular questions of whether components of the synovial inflammatory response are contributing to the loss of sMPC chondrogenic phenotype.

Macrophages are immune cells derived from the monocyte lineage that have been demonstrated to play roles in nearly all aspects of the immune system, tissue remodeling and wound healing [10]. In recent years, the regulation and function of macrophage sub-types has become quite complex, but most agree that there are at least three distinct subtypes; M0 (or inactive), M1 (pro-inflammatory) and M2 (anti-inflammatory) [11]. A number of studies have also demonstrated sub-sets within each of these subtypes that can be distinguished by cell surface receptors and protein secretion. Macrophages are the primary inflammatory custodians of the synovial tissue and can partake in pro- (M1) and anti- (M2) inflammatory activities [12]. To date however, there has been limited characterization of the synovial macrophage; the components of its inflammatory milieu (across patient populations and disease severity), and if these cells/properties regulate/modify sMPC function and/or potential. Early findings from Bondeson et al. [6] attempting to deplete digested OA synovium cultures of macrophages (using anti-CD14 conjugated beads) found reduced levels of cytokines known to degrade cartilage, such as TNFα, IL-1β, IL-6, IL-8, MMP-1 and MMP-3. However, despite such findings, this study did not assess the sMPC population, or changes to its differentiation capacity (if any) following macrophage depletion. A recent study by Fahy and colleagues in 2014 explored this issue, exposing sMPCs to conditioned media (CM) from either digested OA synovium or peripheral blood mononuclear cell-derived M1/M2 macrophages [7]. The authors found that an anti-inflammatory supernatant was more conducive to sMPC chondrogenesis compared to a pro-inflammatory supernatant. Such findings encouraged the present study, with specific interest to assess variations across the patient population.

While the sMPC-macrophage relationship remains undefined in OA, macrophages have demonstrated both promoting and hindering roles on MPC activity in other tissues. For example, tumorigenic MPCs were found to be activated by macrophages (and their inflammatory cytokines) in the tumor site of gastric cancers [13]. Meanwhile, macrophages were also found to be critical regulators of MPC osteogenic differention via STAT3 signalling [14]. Additionally, M2-phenotype macrophages and their associated cytokines supported MPC-graft survival in the infarct site of damaged heart muscles compared to an M1-phenotype macrophage and associated cytokines [15]. Such studies are noteworthy, as they help to illustrate the dynamic MPC-macrophages relationship across normal and diseases states. The goal of the present study was to study endogenous synovial macrophages and their interactions with sMPC chondrogenesis in normal individuals and OA patients using a novel explant system. By manipulating the presence and/or phenotype of synovial macrophages, we hypothesized that the innate chondrogenic capacity of OA sMPCs could be attenuated. Our findings support this hypothesis in patient-specific manner, and also demonstrate a systemic shift in the inflammatory phenotype of synovial tissue itself in response to cytokine stimulation and/or macrophage depletion.

## Methods

### Patients

Patients with clinical and radiographic OA provided synovial membrane biopsies through informed consent during non-emergent knee arthroscopy for meniscal/ligamentous repair at the Peter Lougheed Centre, in Calgary, AB. Synovial membrane biopsies from macroscopically normal knees were obtained from cadavers less the 4 h post-mortem. Tissue donors were received by the Southern Alberta Organ and Tissue Donation Program (SAOTDP), which obtains the medical history of every donor, including current medication, previous history of joint diseases, and other co-morbidities (e.g., cancer, diabetes, inflammatory diseases). All donor knees received X-Ray and macroscopic examination of the joint surfaces. Any abnormalities (cracking, blistering, darkening, abnormal wear) prompted exclusion from the study. All pertinent information is presented in Table 1. The procedures followed were in accordance with the ethical standards of the responsible committee on human experimentation (institutional and national) and with the Helsinki Declaration of 1975, as revised in 2000 and all ethics were approved by the University of Calgary research ethics board.

**Table 1:** Summary of demographic information of donor patients (OA & Normal)

OA Patient	Age	Sex	Procedure	Additional notes
Patient 1	43	M	Right ACL reconstruction	Synovitis observed
Patient 2	30	M	Right ACL reconstruction	Synovitis observed
Patient 3	62	F	Right Knee Arthroscopy for torn lateral meniscus	Patellar tendon OA & on different cancer medication
Patient 4	50	M	Right Knee Arthroscopy & EUA^a^	Advanced (bare bone) OA & candidate for joint replacement
Patient 5	71	M	Left Knee Arthroscopy for torn medial meniscus	Cartilage surface damage & cracked cartilage on tibial plateau
Patient 6	51	M	Right ACL reconstruction	
Patient 7	45	M	Left ACL reconstruction	2 previous failed ACL reconstructions & synovitis
Patient 8	39	M	Left ACL reconstruction	
Normal Samples	Age	Sex		
Normal 1	78	M		
Normal 2	37	M		
Normal 3	54	F		
Normal 4	50	F		

^a^ Examination under anaesthetic (EUA)

### Explant model

**Fig. 1:**
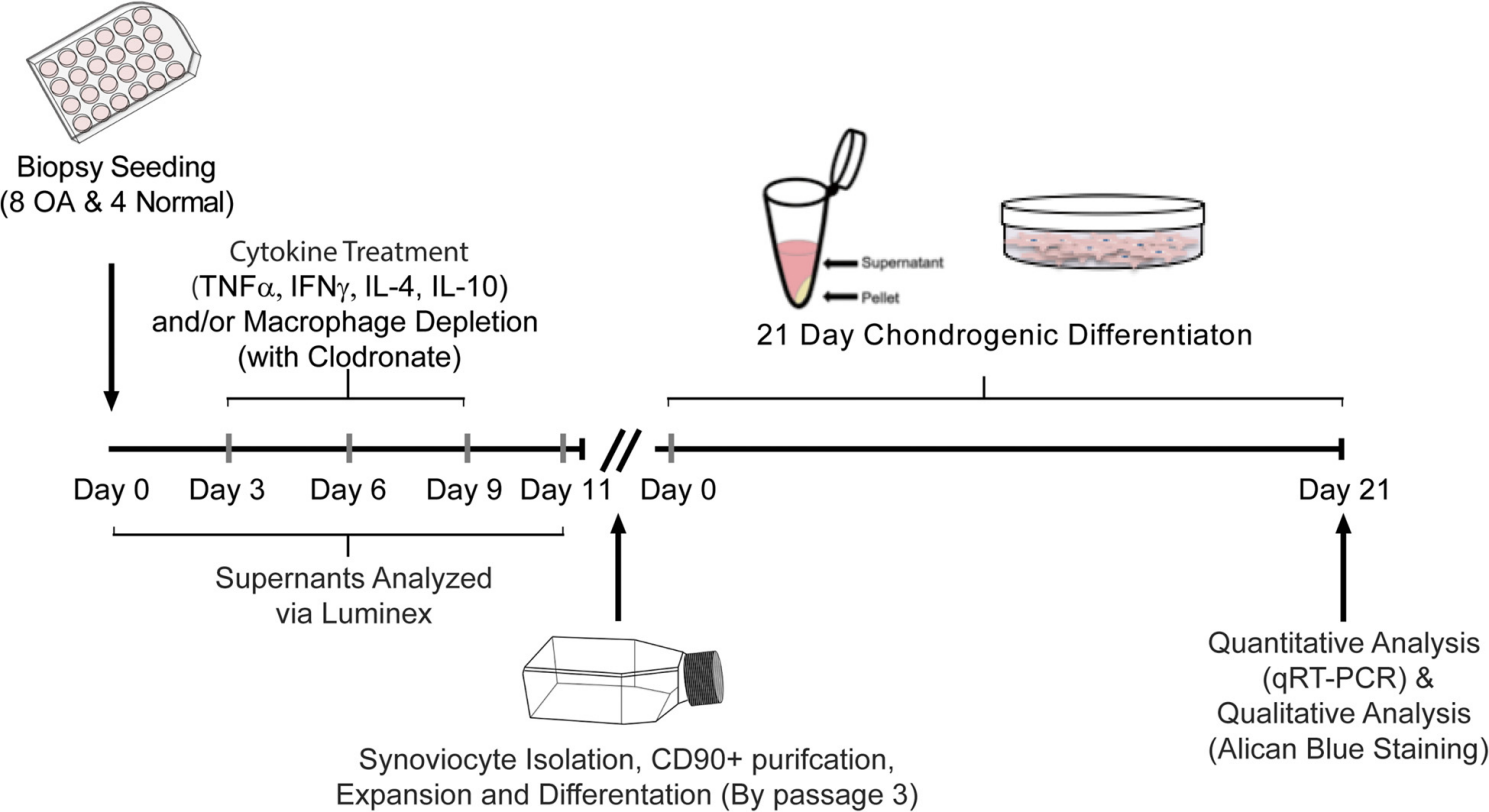
Explant study experimental flow chart

Intact synovial biopsies are a physiologically relevant ex-vivo model to elucidate the sMPC-macrophage relationship in healthy and diseased patients. In this study, upon receipt of synovium (8 OA and 4 normal), biopsy samples were seeded in a 24-well cell culture plate. On days 3, 6, and 9 post-seeding, macrophages within the synovial biopsy samples were subjected to artificial cytokine stimulation and/or depletion using a cocktail of compounds in order to observe how such manipulations affected the sMPC population (Fig. 1). Specifically, 20 ng/mL of pro-inflammatory (IFNγ and/or TNFα) and anti-inflammatory (IL-4 and/or IL-10) cytokines were added because of previously shown roles in macrophage activation/polarization, and articular cartilage degradation [16]. These cytokines were added in the presence or absence of 1000 μM clodronate disodium (Dichloromethylenediphosphonic Acid Disodium Salt – Sigma) in PBS solution. Clodronate disodium, a first generation bisphosphonate, was utilized to selectively deplete macrophages [17–19]. This concentration was utilized based on a dosage analysis from 100, 1000, and 10,000 μM concentrations (analyzed via flow cytometry) which revealed selective macrophage depletion and sMPC preservation at 1000 μM (Table 2). On day 11 post-seeding, outgrown fibroblastic synoviocytes from the OA biopsy samples were collected, purified, expanded in culture as described below in “sMPC culture”. Upon expansion, OA sMPCs from all treatment groups were subjected to 21-day chondrogenesis. Specifically, sMPCs were placed under pellet-culture chondrogenesis in which 50,000 cells were aggregated into pellets prior to differentiation. After 21 days, mRNA was isolated from these pellets and subjected to qRT-PCR.

**Table 2:** Effect of Clodronate treatment on macrophage and sMPC populations in synovium (*N* = 4, all Normal)

Sub-population of interest	% Pre-clodronate treatment	% Post clodronate treatment		
		100 μM	1000 μM	10000 μM
CD68+/CD90- (Macrophage)	84.23 (+/- 6.17)	14.68 (+/- 4.25)	6.58 (+/- 1.98)	15.88 (+/- 3.01)
CD68-/CD90+ (sMPC)	1.68 (+/- 1.78)	1.14 (+/- 1.36)	3.77 (+/- 1.12)	4.47 (+/- 2.12)

### sMPC culture

An outgrowth method was utilized to culture sMPCs [20]. Upon receipt of synovium, three (~3 mm^3^) tissue explants were cut from the biopsy and seeded in a 24-well culture plate and incubated at 37 °C and 5 % CO_2_ with 1 mL of MPC culture media added. The MPC culture media consisted of DMEM F12 (Invitrogen #11965), 10 % Fetal Bovine Serum, 1 % Pen/Strep, 1 % Non-essential amino acids (NEAA), 0.2 % β-mercaptoethanol (all Invitrogen, Carlsbad, CA). Within 11 days post-seeding, outgrown cells were adherent to the plastic and reached 30-40 % confluency (referring to rough percentage of flask surface area covered with cells). At this point, cells were gently dissociated via mechanical stimulation and placed with 5 mL of MPC media in a T-25 cell culture flask. Media was changed every 3 days. After cells reached 70 % confluency, the cells were washed, resuspended, and subjected to magnetic MACs purification entailing (1) hematopoietic lineage depletion (FCGR3A, CD19, CD3E, NCAM1, CD14, GYPA, FCGR3B, ITGA2B) and (2) CD90+ positive selection. Purified cells were then placed in 10 mL of MPC media in T-75 flasks. Media was changed every three days and cells were passaged when 70-80 % confluency was reached. Cells passaged a maximum of 3 times were used for chondrogenic differentiation.

### qRT-PCR

Following chondrogenesis, mRNA (from biological duplicates) was collected using Trizol reagent (Invitrogen) and converted to cDNA using the High Capacity cDNA kit (Applied Biosystems, Carlsbad, CA). The cDNA was probed using pre-validated Taqman primer-sets for human Aggrecan (Acan), Collagen IIa (Col2a), and Sox9. All 3 of these genes encode for critical components of chondrocyte/cartilage formation. Aggrecan is a proteoglycan that forms a major component of the extracellular matrix of articular cartilage. Type II collagen is the base structural protein for articular cartilage. Col2a is the gene that encodes for the synthesis of the pro-alpha1 (II) of type II cartilage. Transcription factor SOX9 is a master regulator chondrocyte differentiation and function. Note: There were biological duplicates in all experiments and each gene of interest was run in well-triplicates during qRT-PCR. Human 18S rRNA was used as the housekeeping gene in all qRT-PCR experiments based on previously validated protocols.

### Flow cytometry

The endogenous cell populations of the synovium were characterized using flow cytometry before and after clodronate treatments in order to determine the most efficacious dosage of clodronate to use for explant studies (Table 2). The synovium was first thoroughly minced and then placed in heat-inactivated FBS containing 1 mg/ml collagenase (Sigma) and digested at 37 °C and 5 % CO2 for 2 h under mild shaking. After this, supernatant was collected using a 70 μm mesh. This supernatant was rigorously washed with PBS and fixed with 4 % paraformaldehyde for 5 min. After washing, fixed cells were then incubated away from light for 1 h with a fluorescent antibodies for CD68 (FITC, EBioscience) and CD90 (APC, EBioscience) prior to flow cytometric analysis on an Invitrogen Attune® Acoustic Focusing Cytometer with Red/Blue Laser Configurations. An identical protocol was followed on Day-12 post-seeding of biopsies samples that were subjected to macrophage depletion via clodronate disodium treatment.

### Luminex

On days 0 and 11 of the sMPC outgrowth phase in the explant study, samples of the normal and OA biopsy supernatant were collected and subjected to inflammatory secretome analysis using Luminex. Multiplexed array technologies allow for multiple concentrations of specific proteins (cytokines and chemokines were used in our study) to be quantified within a single biological fluid sample. This is accomplished in a manner that enables direct comparisons of protein concentration values between samples. In this study, forty-one selected inflammatory proteins were examined. Note: Sample analysis was performed by Eve Technologies (Calgary, AB Canada) using the Milliplex MAP Human Cytokine/Chemokine Panel (Millipore) on a Luminex 100 platform (Luminex Corp., Austin, TX), according to the manufacturer’s instructions. All samples were assayed at least in duplicate and prepared standards were included in all runs. In the analysis, baseline media analyte levels were accounted for.

### Statistical analysis

GraphPad Prism 6 software was utilized to conduct Multiple Comparisons Two-Way ANOVA in all qRT-PCR analysis in which the mean gene expression from each treatment group was compared against the control row and SEM was used to assess the standard deviation of the sampling distribution. Statistical significance was set at *p* < 0.05.

For the Luminex data, all samples were normalized to controls to account for the variance between individuals. Multivariate analysis of variance (MANOVA) was applied to test the difference between samples with and without clodronate, *p* < 0.05 was considered statistically significant. Analysis of variance (ANOVA) with Bonferroni multiple comparison correction was applied to each cytokine to filter out specifically which ones are different between samples with and without clodronate, *p* < 0.0012 was considered statistically significant. Principle component analysis (PCA) was applied and first 9 components (represent >80 % of total variance) were selected for hierarchical clustering. Dendrograms for 4 conditions were drawn based on hierarchical cluster results. SPSS 21 (SPSS, Inc., Chicago IL) was used for Luminx analyses and dendrogram construction.

## Results

### Chondrogenic differentiation analysis

**Fig. 2:**
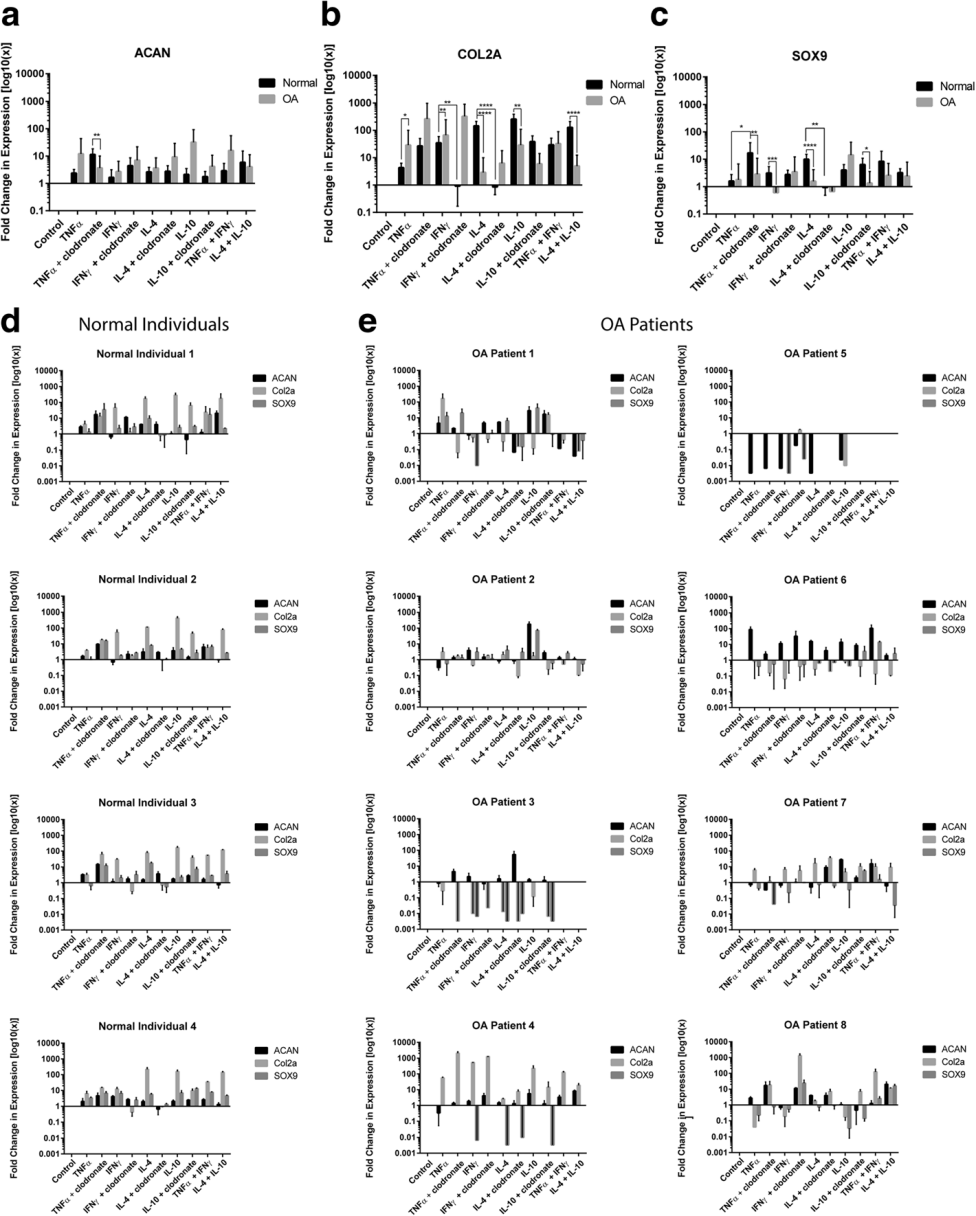
Fold-changes of chondrogenic markers in patient derived sMPCs. Expression levels of Aggrecan (Acan) (**a**), Collagen 2a (Col2a) (**b**), and Sox9 (**c**) were examined with or without cytokine stimulation and/or macrophage depletion (clodronate). All genes were normalized to the housekeeping gene 18 s and the expression levels of each gene is shown relative to untreated (control) sMPCs following 21-day pellet culture chondrogenesis. Fold changes in sMPC from normal individuals (**d**) and OA patients (**e**) were also examined. *p < 0.05, **p < 0.01, ***p < 0.001, ****p < 0.0001

Normal and OA sMPCs that received pro-/anti- inflammatory cytokines and/or 1000 μM clodronate disodium treatment during sMPC outgrowth were subjected to 21-day chondrogenesis via pellet culture. As shown in Fig. 2, pooled gene expression data derived from post-chondrogenic pellet cultures of 4 normal and 8 OA patients revealed that ACAN expression was only significantly different between normal and OA in the TNFα plus clondronate group, while multiple differences were observed in Col2a and Sox9 expression levels both between normal and OA across treatment groups (IFNγ, TNFα, IL-4 or IL-10), and also between the presence or absence of clodronate within normal and OA treatment groups (Fig. 2a-c). Specifically, most of the treatment groups (IFNγ, TNFα, IL-4 or IL-10) resulted in an increased expression of chondrogenic marker expression compared to the control (sMPCs derived from untreated biopsies). IFNγ and IL-4 treatment increased the expression of Col2a in normal sMPCs, and this effect was lost when clodronate was added. This same effect was observed in Sox9 expression with IL-4 treatment, but not IFNγ treatment (Fig. 2c), however, these treatment effects were not observed in OA sMPCs.

Un-pooling and examining gene expression patient by patient revealed more treatment-specific effects in each patient individually (Fig. 2d-e). Overall, it can be observed that the sMPC lines derived from the normal individuals demonstrate a similar gene expression profile across the different treatment groups (IFNγ, TNFα, IL-4 or IL-10) with nearly every treatment resulting in an increase of ACAN, Col2a and Sox9 expression, with the exception of IL-4 plus clodronate that resulted in a reduction of Col2a and Sox9 expression (Fig. 2d). sMPCs derived the 8 OA patients demonstrate a very heterogeneous response across all treatments (Fig. 2e). Specifically, some OA patient derived sMPCs demonstrate a global reduction in chondrogenic gene expression across all or almost all treatment groups (Fig. 2d, OA patients 3 and 5), while other patients demonstrated increases in chondrogenic gene expression across many treatment groups (Fig. 2d, OA patients 2 and 8).

### Inflammatory secretome analysis (Luminex)

**Fig. 3:**
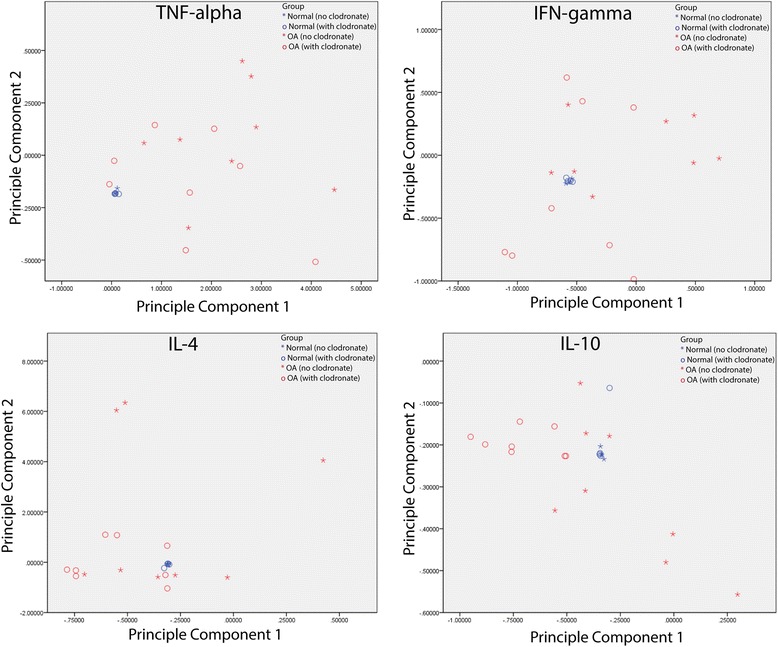
Principle component analysis of cytokines and chemokines secreted from synovial explants. Supernant from 8 OA and 4 normal biopsies was examined using the Luminex platform. Normal biopsies clustered together in all treatment groups (IFNγ, TNFα, IL-4 or IL-10), regardless of the presence or absence of clodronate. OA biopsies demonstrated no discernable pattern with the exception of IL-10 treatment in which all biopsies treated with or without clodronate clustered together

**Fig. 4:**
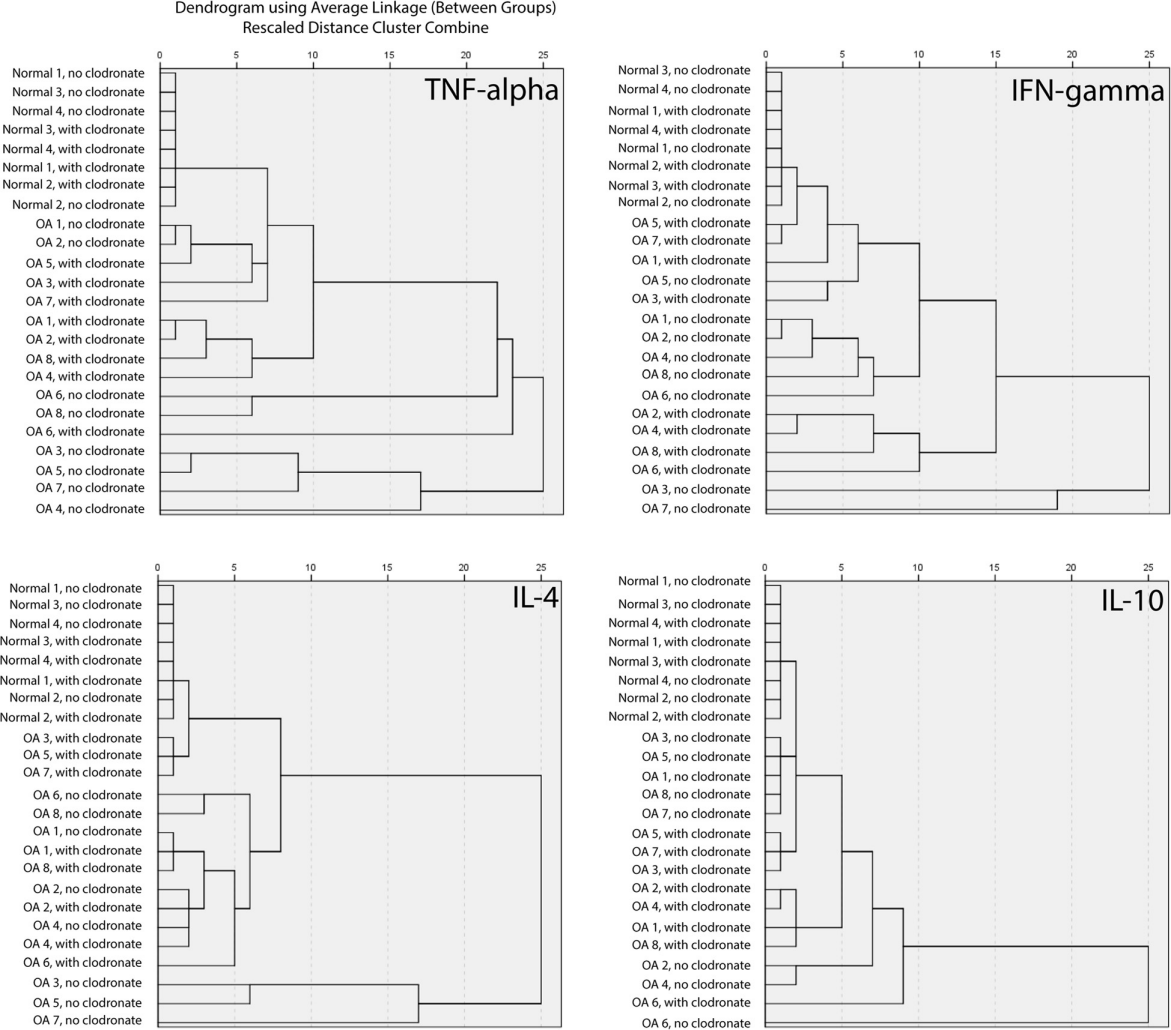
Dendrogram clustering analysis of cytokines and chemokines secreted from synovial explants. Dendrogram analysis of the inflammatory secretome from normal and OA biopsies after cytokine stimulation with or without clodronate treatment demonstrated that all normal biopsies grouped together under all treatment groups, while OA samples clustered on the presence/absence of clodronate

To determine if clodronate treatment had any effect on the cytokines and chemokines produced from synovial explants in the presence of cytokine stimulation, the entire panel of markers (41 analytes) was compared across groups as a complete profile (Table 3). The normal explants demonstrated no differences in the cytokine/chemokine profile after treatment with IFNγ, TNFα, IL-4 or IL-10 in the presence or absence of clodronate (Table 3). However, the inflammatory secretome profile of OA explants was different after clodronate exposure when the explants were stimulated with IFNγ or IL-10, but not TNFα or IL-4 (Table 3). The profiles of the normal and OA explants after IFNγ, TNFα, IL-4 or IL-10 in the presence or absence of clodronate was also examined with principle component analysis (Fig. 3), which demonstrated that all (or most) of the normal samples clustered tightly together under each condition usually surrounded closely by OA samples without clodronate treatment (Fig. 3). In general, the OA profiles were very dispersed compared to the normal profiles and no clear grouping could consistently be observed, with the exception of IL-10 treatment where there is a clear clustering of OA profiles separated by the presence or absence of clodronate treatment (Fig. 3). To analyze the groupings of profiles in more detail, dendrogram clustering analysis was undertaken on the inflammatory secretome profiles of all samples under each treatment group (Fig. 4). As in the PCA, it is clear that all normal samples cluster together irrespective if clodronate was added or not. Furthermore, it can be observed that OA profiles group together based on if clodronate was added under each treatment group (IFNγ, TNFα, IL-4 or IL-10) (Fig. 4).

**Table 3:** Comparison of normal and OA secreted inflammatory factors in the presence or absence of clodronate after cytokine stimulation

	INFγ	TNFα	IL-4	IL-10
Normal (*N* = 4)	*p* = 0.248	*p* = 0.088	*p* = 0.851	*p* = 0.619
OA (*N* = 8)	*p* = 0.016*	*p* = 0.094	*p* = 0.130	*p* = 0.044*

*significance was set at *p* < 0.05

The inflammatory secretome profile of each treatment groups was also examined at the individual cytokine/chemokine level to determine which proteins were contributing the differences observed in the overall profiles (Table 4). Interestingly, although no differences were observed in the complete profiles of the normal samples under any conditions, when the individual proteins were examined it was found that MCP-1/CCL2 expression was different in the presence of clodronate after IFNγ, TNFα, IL-4 or IL-10 treatment (Table 4). In the OA samples, a number of proteins under each condition (IFNγ, TNFα, IL-4 or IL-10) were determined to be significantly different in the presence vs. absence of clodronate (Table 4), however, MCP-1/CCL2 was not observed to be different in any OA profiles within any treatment groups.

**Table 4:** Differentially expressed cytokines in clodronate vs. no-clodronate conditions

Normal (*N* = 4)				OA (*N* = 8)			
INFγ	TNFα	IL-4	IL-10	INFγ	TNFα	IL-4	IL-10
MCP-1	MCP-1	MCP-1	MCP-1	IL-10	Eotaxin	IL-15	TNF-β
*p* = 6.2x10^-5^	*p* = 3.1x10^-5^	*p* = 9.8x10^-5^	*p* = 6.7x10^-6^	*p* = 2.0x10^-6^	*p* = 4.2x10^-8^	*p* = 5,6x10^-6^	*p* = 4.6x10^-5^
				VEGF	MIP-1α	Fit-3 L	EFG
				*p* = 3.6x10^-6^	*p* = 3.6x10^-5^	*p* = 0.0002	*p* = 5.4x10^-5^
				TGFα	MIP-1β	VEGF	MIP-1α
				*p* = 9.0x10^-6^	*p* = 0.0008	*p* = 0.0005	*p* = 0.0002
				GRO			IL-4
				*p* = 2.0x10^-5^			*p* = 0.0003
				RANTES			IL-7
				*p* = 4.4x10^-5^			*p* = 0.0005
				Eotaxin			IL-12p40
				*p* = 8.2x10^-5^			*p* = 0.0005
				MCP-3			MCP-3
				*p* = 8.6x10^-5^			*p* = 0.0009
				IL-5			
				*p* = 0.0001			
				EGF			
				*p* = 0.0002			

After Bonferroni correction *p* < 0.05/41 = 0.0012

## Discussion

The aim of the present study was to elucidate the relationship between synovial macrophages and synovial MPCs. This was prompted by literature findings that macrophages are drivers of synovitis and cartilage loss in OA, and are thus likely candidates to regulate sMPC chondrogenic differentiation [6, 7, 16, 21]. Other studies have hypothesized and reported that an M1-macrophage phenotype and its mediators have anti-chondrogenic effects [6, 7]. Despite these findings, the present study is the first of its kind (to our knowledge) to show a heterogeneous and patient-specific response following attenuation of macrophages state (via cytokine stimulation or depletion) on sMPC chondrogenesis.

Using a synovial explant model that was to be subjected to pro- and anti-inflammatory cytokine stimulation and/or macrophage depletion, we first confirmed the presence of endogenous macrophages using flow cytometry and immunofluorescent staining (Table 2 and Additional file 1: Figure S1). Post 21-day sMPC pellet-culture chondrogenesis, qRT-PCR was used to quantify the differentiation process. Pooled sMPC chondrogenic gene expression data from 8 OA patients and 4 normal individuals revealed a non-treatment specific up-regulatory response in the levels of Acan, Col2a, and Sox9 expression (Fig. 2) when the explant (not the purified sMPCs) had been treated with IFNγ, TNFα, IL-4 or IL-10 in the presence or absence of clodronate. The nature of these findings prompted closer inspection of the data on an individual patient-specific basis.

Assessment of the qRT-PCR findings of all 8 OA patients and 4 normal individuals revealed a heterogeneous response to all treatments in the OA patient derived sMPCs, in which macrophages promoted, inhibited, or had no apparent effect on sMPC chondrogenesis. For example, in OA Patient 1, sMPC Col2a expression was increased following TNFα treatment compared to TNFα treatment alongside macrophage depletion (clodronate), while, in contrast, sMPCs from OA Patient 4 demonstrated an increased in Col2a expression in the presence of TNFα and clodronate. Patient 3 and 5 demonstrated a near global decrease in chondrogenic gene expression across all treatment groups, while sMPCs from patients 1, 7 and 8 demonstrate a wide variety of responses to all treatment groups (Fig. 2) apparently irrespective of macrophage presence. Overall, these varying treatment responses illustrate the heterogeneity between the sMPCs of OA patients and reinforce the idea that OA is a spectrum disease with numerous avenues (injury, metabolic, idiopathic) that can lead to a clinical diagnosis [22]. Taken together, it is difficult to interpret the significant differences observed in the pooled data because of the wide and varied response observed within the 8 OA patient samples. This could speak to differences in age, sex and/or severity of disease in the patients, but without a larger sample size it would be inappropriate to stratify and comment on these variables.

The changes in the normal and OA explant inflammatory secretome profiles of during the 12-day treatment phase provide complementary data to the qRT-PCR analysis. These findings shed light on the response of normal and OA synovial tissue to cytokine stimulation (IFNγ, TNFα, IL-4 or IL-10) in the presence or absence of macrophage depletion (clodronate). Interestingly, in normal synovial samples, no profile level changes (all 41 analytes compared together) were observed within or between treatment groups, however, at the individual protein level, it was observed that MCP-1/CCL2 was differentially expressed in under all treatment effects. Our group has previously implicated MCP-1/CCL2 in the loss of chondrogenic capacity of synovial MPCs [21]. In that study we were able to demonstrate that MCP-1/CCL2 doesn’t impede the proliferation MPCs, but only decreased their chondrogenic capacity. Since MCP-1/CCL2 was the only protein that appeared to be regulated by cytokine stimulation with macrophage depletion, coupled with the observation that only a very few treatments reduced the chondrogenic marker expression in normal sMPCs; suggests that the normal synovial explants may have the ability the ‘buffer’ the sMPCs against cytokine stimulation and this may not dependent on the presence of synovial macrophages. Additionally, these results seem to suggest that cytokine stimulation enhances the chondrogenic differentiation of the synovial MPCs. While numerous previous studies have demonstrated that pro-inflammatory cytokines (e.g. TNFα, IL-1β) inhibit the chondrogenic capacity of stem cells [23, 24], it is important to recognize that in the current study, the purified MPCs were never exposed directly to the cytokines, instead the intact synovial biopsies were exposed the cytokines, the cytokines were then removed and MPCs were then derived, purified and differentiated. This could suggest, that while certain cytokines can regulate the capacity of stem cells/MPCs, that when the cells are still within their niche, interaction with the inflammatory micro-environment and other tissue resident cells can lead to distinct outcomes.

The analysis of the OA patient inflammatory secretomes, demonstrated distinct responses compared to the normal explants. Specifically, a number of cytokines and chemokines were differentially expressed based on cytokine stimulation and the state of macrophages in the samples. While there was no single protein that was found across all treatment groups, MCP-3, MIP-1α, and VEGF were all observed in half of the treatment groups. Interestingly, IL-10 was observed in the IFNγ treatment group, while IL-4 was observed to be differentially regulated in the presence of IL-10. Overall, it would appear that OA synovium may no longer have the ability to ‘buffer’ the cytokine stimulation and that effect appears to be regulated by the presence of synovial macrophages. These findings lend support to the idea that the macrophages may have a role to propagate and sustain cytokines being secreted within the synovium, as proposed in the cytokine theory of OA pathogenesis [25–28]. When the inflammatory secretomes of all the samples were analyzed by dendrogram clustering analysis, it was observed that the normal samples always grouped together regardless of treatment group, or if clodronate was present or absent (Fig. 4). Interestingly, the OA samples appeared to group together based on if clodronate was present or absent, however, the same samples didn’t always group together under each treatment group (IFNγ, TNFα, IL-4 or IL-10). For one specific example; samples from patient 1 and 8 without clodronate group together in the presence of TNFα, IL-4 and IL-10, but don’t group together after treatment with IFNγ. This suggests that explants from different patients respond differently to specific cytokines and that this is at least partially dependent on the presence of macrophages in that synovial explant. While a sample size of eight is not large enough to make any definitive conclusions, this could be part of the reason that OA patients demonstrate a wide response to anti-cytokine treatments [29, 30]

Overall, inflammatory secretome data from OA and normal patients reveal a pathology-dependent response, where normal patients did not display observable levels of any analytes (except for MCP-1/CCL2) following cytokine treatments in the presence or absence of macrophages, while OA patient synovium reacted both to the cytokine stimulation and macrophage depletion. Such findings from the normal patients maybe reflective of state of synovial homeostasis in their joints that is ‘buffered’ to changes in the microenvironment. These findings are consistent with previous studies, which have suggested that a healthy synovial joint can maintain a balanced microenvironment [31]. It would be of interest to find exactly when the normal synovium loses the ability to ‘buffer’ cytokine stimulation with the onset and progression of OA. This would most likely require a pre-radiographic and pre-symptomatic patient population that would be difficult to characterize. Additionally, since the normal synovial ‘buffering’ ability did not appear to be solely dependent on the presence of macrophages, it would be of interest to elucidate exactly which cell population(s) are either: providing this capacity in normal tissues, or alternatively, are responsible for the dramatic changes in inflammatory response observed in OA tissues.

This study was not without limitations. The most salient of which is the number of enrolled patients (8 OA and 4 normal). Additionally, despite stringent inclusion/exclusion criteria, the spectrum of disease severity amongst the OA samples was uncontrolled for in this study, and was likely reflected (to some extent) in the degree of heterogeneity observed in the qRT-PCR data and Luminex. Future studies will seek to increase patient enrolment such that early, mid, and late stage OA cohorts could be assessed separately. Beyond this, our study did not assess how the differentially regulated analytes in the OA explant supernatant in response to the cytokine treatments would have ultimately affected the chondrogenic phenotype of sMPCs thereafter. These may serve as novel targets to study, as they may be implicated in the sMPC-macrophage relationship.

## Conclusion

Using a synovial explant model, we have shown that the chondrogenic capacity of sMPCs can be regulated (positively or negatively) by modifying the endogenous synovial macrophage population (via cytokine stimulation and/or depletion). This study has also revealed that the inflammatory phenotype of the OA synovium itself can be responsive to changes in the macrophage population. These findings lend support to the idea that macrophages are intricately involved in sMPC chondrogenesis and are therefore an important cell type for further investigation, and future therapy development.

## Additional file

Additional file 1: Figure S1. Immunofluorescent examination of synovial explants with or without clodronate treatment. Whole-mount immunofluorescent staining of CD68+ Macrophages (FITC). Isolated synovial biopsies before 12-Day sMPC outgrowth phase (A-B). Isolated synovial biopsies after 12-Day sMPC outgrowth phase where 1000 μM clodronate disodium [CLOD (+)] treatment on days 3,6, & 9 post seeding. (TIF 15841 kb)

## Abbreviations

ACAN: aggrecan
ANOVA: analysis of variance
CM: conditioned media
COL2a: collagen IIa
FBS: fetal bovine serum
M1: pro-inflammatory phenotype
M2: anti-inflammatory phenotype
MCP-1: monocyte chemoattractant protein -1
MMP: matrix metalloprotease
MPC: mesenchymal progenitor cell
NEAA: non-essential amino acids
OA: osteoarthritis
PBMC: peripheral blood mononuclear cells
PBS: phosphate buffer solution
Pen: penicillin
qRT-PCR: quantitative real time polymerase chain reaction
SAOTDP: Southern Alberta organ & tissue donation program
sMPC: synovial mesenchymal progenitor cell
