# Supplementary figures and images for: Characterizing heterogeneity in the response of synovial mesenchymal progenitor cells to synovial macrophages in normal individuals and patients with osteoarthritis
Source: J Inflamm (Lond). 2016 Apr 6;13:12. doi: 10.1186/s12950-016-0120-9 (PMC4823907; doi:10.1186/s12950-016-0120-9)

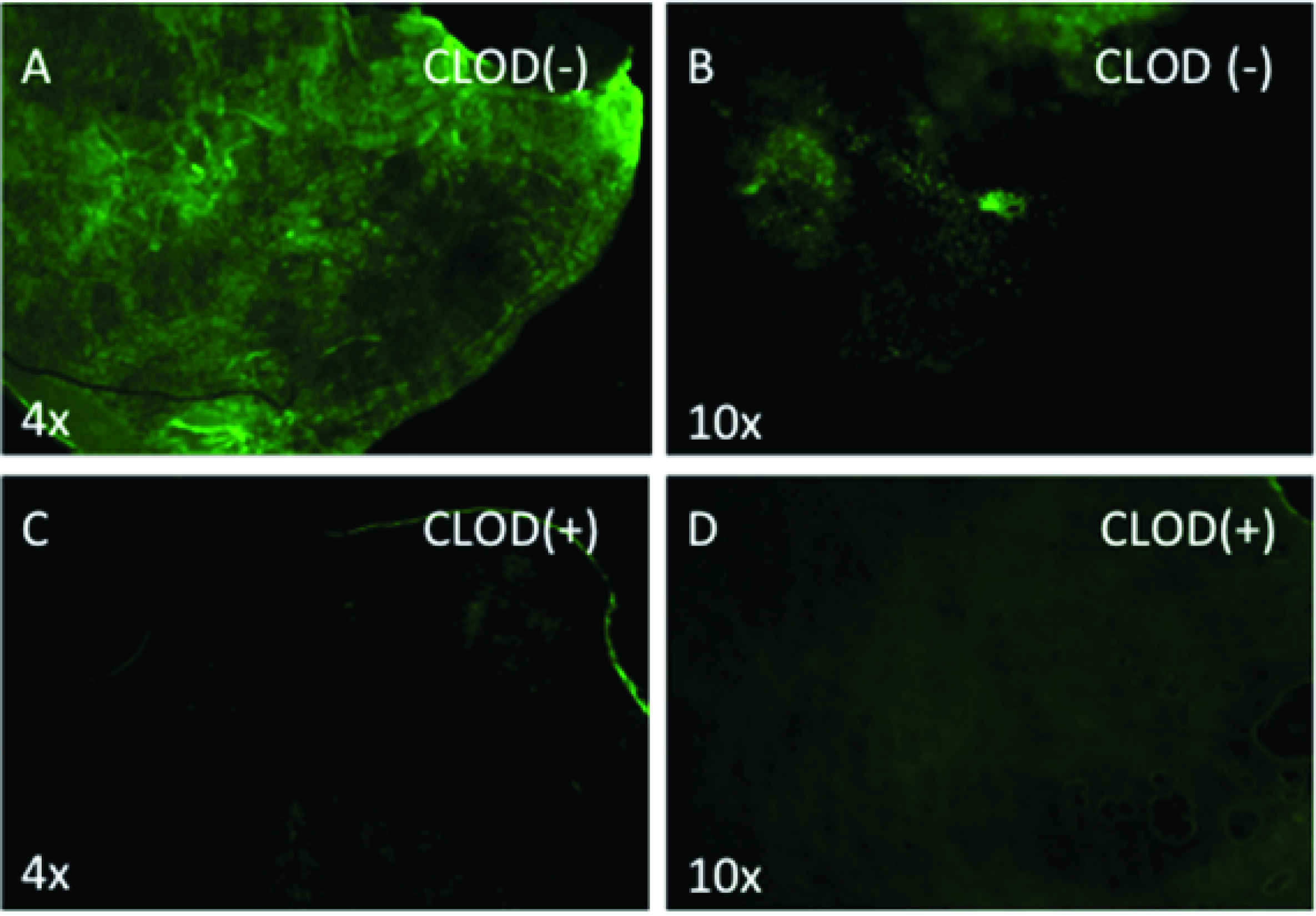

Supplement: Additional file 1: Figure S1. — Immunofluorescent examination of synovial explants with or without clodronate treatment. Whole-mount immunofluorescent staining of CD68+ Macrophages (FITC). Isolated synovial biopsies before 12-Day sMPC outgrowth phase (A-B). Isolated synovial biopsies after 12-Day sMPC outgrowth phase where 1000 μM clodronate disodium [CLOD (+)] treatment on days 3,6, & 9 post seeding. (TIF 15841 kb) [file 12950_2016_120_MOESM1_ESM.tif]
